# Fatty acid profiles of great tit (*Parus major*) eggs differ between urban and rural habitats, but not between coniferous and deciduous forests

**DOI:** 10.1007/s00114-016-1381-0

**Published:** 2016-06-14

**Authors:** Alejandra Toledo, Martin N. Andersson, Hong-Lei Wang, Pablo Salmón, Hannah Watson, Graham C. Burdge, Caroline Isaksson

**Affiliations:** 1 Department of Biology, Lund University, Lund, Sweden; 2 Department of Life Science, University of Roehampton, London, UK; 3 Academic Unit of Human Development and Health, Faculty of Medicine, University of Southampton, Southampton, UK

**Keywords:** Anthropogenic, Development, Nutrition, Incubation, Maternal effects, Polyunsaturated fatty acids

## Abstract

Early-life nutrition is an important determinant of both short- and long-term performance and fitness. The avian embryo develops within an enclosed package of nutrients, of which fatty acids (FA) are essential for many aspects of development. The FA composition of yolk depends on maternal nutrition and condition prior to egg formation, which may be affected by the external environment. To test if maternal environment affects yolk FA composition, we investigated whether the FA composition of great tit (*Parus major*) egg yolks differed between urban and rural habitats, and between deciduous and coniferous habitats. The results reveal differences in FA composition between eggs laid in urban and rural habitats, but not between eggs from the coniferous and deciduous habitats. To a large extent, this difference likely reflects dietary differences associated with urban habitats rather than dominating vegetation type. Specifically, urban yolks contained lower proportions of both ω-3 and ω-6 polyunsaturated FAs (PUFA), which are important for chick development. We also found a positive association between the proportion of saturated fatty acids and laying date, and a negative association between the proportion of ω-6 PUFA and clutch size. Given that urbanization is expanding rapidly, future studies should investigate whether factors such as anthropogenic food in the urban environment underlie these differences and whether they impair chick development.

**Electronic supplementary material:**

The online version of this article (doi:10.1007/s00114-016-1381-0) contains supplementary material, which is available to authorized users.

## Introduction

Urbanization is increasing throughout the world at the expense of natural habitats, imposing changes and challenges for wild animals (Marzluff et al. 2001). The main sources of stress that affect urban animals include habitat degradation, poor quality diet, chemical pollution, artificial night light, noise, pathogens, and local climatic changes (reviewed in Isaksson 2015). Even though some species exhibit increased population densities and winter survival in urban areas (Boutin 1990; Marzluff et al. 2001; Chamberlain et al. 2009), species diversity is usually lower and population demographics often unknown (Marzluff et al. 2001). In addition, several urban stressors have negative effects on physiological health markers among urban wildlife (Isaksson 2015). To understand the impacts of urbanization, it is important to assess if and how individuals from urban and rural populations differ in their underlying physiology; such differences can have implications for development and health (Miller and Hobbs 2002; Isaksson 2015).

One key factor that strongly influences survival and performance of birds is the availability of food. Anthropogenic sources of foods are abundant in cities, e.g., at bird feeding tables and in garbage bins (Jones and Reynolds 2008). Indeed, supplementary food affects avian breeding in a number of ways, including advancement in the timing of breeding (Schoech 1996; Harrison et al. 2010), shorter incubation period, decreased hatching success, clutch and brood size (Harrison et al. 2010), decreased time for raising chicks to fledging, shorter interval between clutches (Verboven et al. 2001), as well as carry-over effects such as delayed laying date the following year (Grieco et al. 2002). Supplementary foods such as sunflower seeds and bread have a different nutritional composition compared with foods that are naturally available to birds, and one of the important nutritional aspects that differs between natural and human-provided foods is the composition of fatty acids (FA; Andersson et al. 2015; Isaksson et al. 2015).

FAs are essential for many physiological processes throughout the life of an animal, particularly so during the early life stages, when tissues and organs grow and develop (Sanders 1988; Maldjian et al. 1996). In birds, the transfer of FAs from the female to the egg yolk is mainly influenced by maternal nutrition as well as age and genetic background (Stadelman and Pratt 1989; Noble and Cocchi 1990). Thus, the embryo development depends on maternal nutrition and the allocation of nutrients to the yolk. A large proportion of the yolk FAs is used to satisfy the energy demands of the developing embryo (Noble and Cocchi 1990). In addition, several embryonic tissues require specific omega-3 (ω-3) and omega-6 (ω-6) polyunsaturated fatty acids (PUFA) in order to develop adequately. For instance, phospholipids in the cell membranes of the brain, retina, and skeletal muscles have a high demand for docosahexaenoic acid (DHA) because of their excitable properties (Neuringer et al. 1988; Anderson et al. 1989; Maldjian et al. 1996; Speake et al. 1996; Mitchell et al. 1998; Salem et al. 2001; Speake and Wood 2005). Another example of a crucial FA is arachidonic acid, which is important for regulation of both brain function and heartbeat (Hohl and Rosen 1987; Katsuki and Okuda 1995; Pavoine et al. 1999). Suboptimal assimilation of these FAs may reduce the viability of the embryo or constrain the development of embryos and nestlings.

There is abundant literature concerning variation in the yolk FA profile of poultry (Surai and Speake 2008, and references therein). However, only little is known about natural variation in yolk FA composition among wild bird populations (Bourgault et al. 2007), and it is unknown if factors in the urban environment affect yolk composition. Hence, using two pairs of great tit (*Parus major*, L. 1758) populations, we investigate whether two ecological factors—degree of urbanization and dominating vegetation type—affect the FA composition of the egg yolk. Specifically, we compared the FA composition of yolks from urban parks versus those from a rural forest, as well as from plots of coniferous versus deciduous forest habitats. Great tit plasma FA composition differs between these two habitat pairs in both adults and nestlings, which appears to be associated with differences in food availability, including foods provided by humans (Andersson et al. 2015; Isaksson et al. 2015). In addition, Bourgault et al. (2007) found that a few yolk FAs in the closely related blue tit (*Cyanistes caeruleus*, L. 1758) differed between deciduous and evergreen forests. Based on these previous findings, we hypothesize that great tits from the two habitat pairs would exhibit variation in FA composition also in the yolk. More specifically, since supplementary food such as seeds and nuts is likely to be more readily available during egg formation for birds in urban habitats, we predict that urban eggs would have higher relative levels of oleic acid (a MUFA), and linoleic acid (an ω-6 PUFA), due to their high abundance in these food sources (Beare-Rogers et al. 2001; Becker 2008; Andersson et al. 2015). During the period of egg formation, the great tits in the study populations are mainly granivorous; however, females from forests (regardless of the forest type) are likely to have more access to food sources containing ω-3 PUFAs, such as overwintering insect pupae (Perrins 1991; Gosler 1993; Andersson et al. 2015; Isaksson et al. 2015). Thus, we predict that eggs from the forest will contain a higher proportion of ω-3 PUFA compared to eggs from the urban habitat.

In addition, both urbanization level and forest type are known to affect great tit clutch size and fledging success. Specifically, rural and deciduous habitats are associated with higher reproductive success compared with urban and coniferous habitats, respectively (Perrins 1991; Chamberlain et al. 2009). However, whether yolk FA composition is associated with habitat-related differences in clutch size is unknown. Thus, we tested whether yolk FA composition is associated with clutch size and also the egg’s number in the laying sequence. We predict that FAs that are limited in the diet, such as ω-3 PUFAs, would be present at lower proportions in eggs from larger clutches and those laid later in the laying sequence.

Finally, using eggs artificially incubated for different periods of time, we tested whether the yolk FA proportions were different depending on incubation time and whether such variation differed between the urban and rural habitats, suggesting different availability of FAs to the embryo.

## Material and methods

### Study sites

Detailed characteristics of the field sites have been described in previous publications (Isaksson 2013; Isaksson et al. 2013, 2015; Andersson et al. 2015). All sites support nest box breeding populations of great tits. The site used to investigate potential differences between deciduous and coniferous habitats was Bagley Woods, Oxfordshire (51° 42′ N, 5°37′ W), United Kingdom (UK), which is a 250-ha mixed matrix woodland with plantations of either strictly coniferous trees (mainly pine, *Pinus* spp., and larch, *Larix* spp.) or deciduous trees (mainly oak, *Quercus* spp.). Eggs were sampled in both habitat types (coniferous: *n*_plots_ = 3; deciduous: *n*_plots_ = 3); for map see Isaksson et al. (2013). These habitat-plots are the exact same ones as sampled by Isaksson et al. (2015) during the same year for analysis of FA levels in plasma of both nestling and adult great tits. The urban versus rural comparison was performed in Swedish populations. Eggs from the urban habitat were collected from several parks (Andersson et al. 2015) within the city limits of Malmö (55° 35′ N, 12° 59′ E), i.e., the third largest city of Sweden with approximately 300,000 inhabitants. Rural eggs were collected from the forest of Vomb (approximately 42 km NE of Malmö, 55° 40′ N, 13° 31′ E), which is sparsely populated by humans. These sites are the exact same ones as used by Andersson et al. (2015) the previous year (2013) for analyses of FA profiles of adult great tit plasma. Supplemental feeding of birds with seeds and nuts was observed in the urban parks, but not in the forest of Vomb; thus, the variety of food items available to great tits was different between the urban and rural populations. Both the urban and rural habitats are located in the province of Scania in southernmost Sweden (for satellite images and urbanization categorization, see Andersson et al. 2015). The vegetation in the urban city parks is dominated by deciduous trees (mainly *Fagus*, *Quercus*, *Betula*, and *Salix* spp.), but also includes some conifers (*Pinus*, *Picea*, and *Larix* spp.), along with larger open managed grassland and the urban influences of paved roads and buildings. The rural site at Vomb comprises a mix of conifer and deciduous (mostly *Betula* and *Quercus* spp.) trees, but with scots pine, *Pinus sylvestris*, as the most abundant species. Because both the urban and rural habitats comprise heterogeneous mixtures of deciduous and conifer trees, the vegetation composition in terms of percentage of deciduous trees and canopy cover (0, 20, 40, 60, 80, or 100 %; the 0 % category lacking for canopy cover) was monitored at a 30-m radius around each nest box from which eggs were collected.

### Fieldwork

All national regulations and ethical guidelines were followed. Eggs were collected with permission from Natural England and the Swedish Environmental Protection Agency. In addition, ethical permission for sacrificing embryos was granted by the Swedish Board of Agriculture (Dnr 454 12:1).

In the coniferous and deciduous habitats in the UK, eggs were collected between the 13th and 28th of April 2010, and in the rural and urban habitats in Sweden between the 13th and 26th of May 2014. Breeding stage was monitored every second day from the first signs of nest construction. When finding eggs, they were marked with a non-toxic pen; the following day, the newly laid (i.e., unmarked) egg was collected. One egg was collected per nest; in the UK, it was between the 2nd and 5th egg in the laying sequence (median = 3; 2nd (*n*_deciduous_ = 8, *n*_coniferous_ = 7), 3rd (*n*_deciduous_ = 6, *n*_coniferous_ = 5), 4th (*n*_deciduous_ = 7, *n*_coniferous_ = 2), and 5th (*n*_deciduous_ = 3, *n*_coniferous_ = 6)). In Sweden, it was always the third egg in the laying sequence that was collected (*n*_urban_ = 15, *n*_rural_ = 15). In total, 44 eggs were collected for the coniferous-deciduous comparison and 30 eggs for the urban–rural comparison. The yolks from the UK were separated from the albumen and immediately stored at −80 °C until biochemical analysis. The Swedish eggs were stored at 12 °C for no more than 3 days until the start of artificial incubation (incubators: Ruvmax, Stockholm, Sweden). Eggs were incubated at 37 °C for a period of either 24 h (*n*_urban_ = 5, *n*_rural_ = 5), 6 days (*n*_urban_ = 5, *n*_rural_ = 5), or 12 days (*n*_urban_ = 5, *n*_rural_ = 5). Following incubation, embryos were terminated and stored at −80 °C until yolk separation and analysis.

### Fatty acid extraction and quantification

The FA composition of yolk from the British eggs was determined as described in Burdge et al. (2000) and Isaksson et al. (2015). Briefly, a portion of the yolk was powdered in liquid nitrogen and approximately 10 mg of the powder was used for extraction using chloroform and methanol (2:1 *v*/*v*). FAs in the plasma were converted to FA methyl esters (FAMEs) by reaction with acidified methanol (Burdge et al. 2000). FAMEs were measured using BPX70 fused silica capillary column (30 m × 0.25 mm × 0.25 μm) on an Aglient 6890 gas chromatograph (GC) equipped with flame ionization detection (Burdge et al. 2000). FAMEs were identified by their retention times relative to standards and quantified using Chemstation software (Agilent).

For the Swedish samples, the FA extraction followed the methods described in Andersson et al. (2015), starting from approximately 5 mg yolk. Base methanolysis was carried out to transform the FAs into FAMEs. The samples were analyzed on an Agilent 6890 GC equipped with an HP-88 capillary column ((88 %-cyanopropy)aryl-polysiloxane; 30 m, 0.25 mm id, *d*_*f*_ 0.20 μm; Agilent), and an Agilent 5975 mass spectrometry detector. Analyses and quantification of chromatograms were performed using Chemstation software (Agilent). FAMEs were identified by comparing mass spectra and retention times with those of synthetic standards (Supelco 37-Component FAME Mix, Sigma-Aldrich).

### Data handling and statistical analysis

The proportion of each FA was calculated by dividing the peak area by the sum of the peak areas of all FAs in each individual sample. The proportions of all individual FAs within a certain chemical class of FA were then combined to obtain relative levels of total SFA, total MUFA, total ω-3 PUFA, total ω-6 PUFA, and total PUFA (i.e., ω-3 and ω-6 combined). In addition, the within-egg ratio between total ω-6 PUFA and total ω-3 PUFA was calculated. In previous publications, we have analyzed each FA that comprised >1 % of the total fatty acid content individually, because most of the individual FAs, both major and minor ones, revealed clear differences between habitats or, in particular, between seasons (Andersson et al. 2015). However, in the current dataset, obvious differences were mostly found among the most abundant FAs, and they correlated well to the abundance of the particular FA class to which they belonged. Thus, for the sake of simplicity and to reduce the risk of type I errors (false positives) due to multiple testing, we limited the statistical analyses to the five classes of FAs (i.e., total SFA, total MUFA, total PUFA, total ω-3 PUFA, and total ω-6 PUFA), as well as the total ω-6/ω-3 ratio. The results for individual FAs are presented using descriptive statistics only. Before statistical analysis, all proportions were logit-transformed (log10(y/[1-y])), as recommended for proportional data (Warton and Hui 2011), and the ratio between total ω-6 PUFAs and total ω-3 PUFAs was log10-transformed.

Since the sampling and biochemical analysis methods differed between the Swedish and British eggs, and because populations in the different countries are geographically well separated and were sampled during different years, we performed the statistical models separately for the two datasets. We do not make any statistical comparisons between eggs from the two countries. The five FA proportions (total SFA, total MUFA, total PUFA, total ω-3 PUFA, and total ω-6 PUFA), along with the total ω-6 to total ω-3 ratio, were used as response variables in both data sets. The analysis (general linear models, GLMs) of the British yolks included “habitat type” (coniferous or deciduous) as a fixed factor, and “laying order,” “laying date,” and “clutch size” as covariates.

Two different statistical tests were performed using the Swedish dataset. Firstly, a similar GLM as for the British dataset was performed, using habitat (rural and urban) as a fixed factor, and laying date and clutch size as covariates. In addition, we included artificial “incubation time” (incubation for 1, 6, or 12 days, i.e., different developmental stages) as a fixed factor. We maintained all the above models the same regardless of the significance levels of main factors and interactions, to facilitate comparisons between different FAs within each habitat pair. Secondly, when testing the interaction between habitat and incubation time, we simplified the GLMs (due to the limited sample size) by removing the two covariates (i.e., clutch size and laying date). In addition, the vegetation composition data of urban and rural habitats were used in correlation analyses to test for possible associations between each of the FA groups and the percentage of deciduous trees and canopy cover within the territory. These correlations were run for the two habitats separately to isolate the effect of vegetation composition from other factors that differ between urban and rural habitats.

Levene’s and Shapiro–Wilk tests were used for analysis of homoscedasticity and normality of residuals, respectively. Model assumptions were met in most cases; some variances were marginally different between groups, especially across incubation periods. However, given the robustness of GLMs for deviations from normality, we decided to use GLMs for all FA analyses, which also ease comparisons across FA groups. The α-level was set at 0.05. For all analyses, denominator degrees of freedom for fixed effects were calculated using the Satterthwaite approximation. Statistical analyses were performed with R 3.1.2 (R Core Team 2014) using the lmerTest (Kuznetsova et al. 2014) package.

## Results

In total, 18 FAs were identified and quantified in great tit egg yolks. The relative levels varied substantially between different FAs, with the most abundant one (oleic acid) contributing close to 40 % of the total FA content, whereas several FAs were present at less than 1 % of total FA, with certain FAs not even detected in some habitats (Table 1).

**Table 1:** Relative abundance (% of total fatty acid content) and classification of the fatty acids identified in great tit egg yolks in the four habitats

Trivial name	C:Dn-x	Fatty acid class	UK		Sweden	
			Deciduous habitat	Coniferous habitat	Rural habitat	Urban habitat
			Mean % ± SE	Mean % ± SE	Mean % ± SE	Mean % ± SE
			(*n* = 24)	(*n* = 20)	(*n* = 15)	(*n* = 15)
Myristic acid	14:0	SFA	0.93 ± 0.17	1.45 ± 0.12	0.64 ± 0.03	0.70 ± 0.13
Palmitic acid	16:0	SFA	23.72 ± 0.51	23.4 ± 0.44	22.94 ± 0.35	22.38 ± 0.96
Stearic acid	18:0	SFA	8.54 ± 0.16	8.42 ± 0.17	7.84 ± 0.14	8.58 ± 0.23
Palmitoleic acid	16:1n-7	MUFA	2.30 ± 0.18	2.52 ± 1.18	1.54 ± 0.08	1.32 ± 0.28
*cis*-Vaccenic acid	18:1n-7	MUFA	1.53 ± 0.06	1.62 ± 0.06	2.92 ± 0.12	2.86 ± 0.17
Oleic acid	18:1n-9	MUFA	38.15 ± 0.77	36.43 ± 0.66	31.23 ± 0.71	39.37 ± 1.78
Eicosenoic acid	20:1n-9	MUFA	ND	ND	0.47 ± 0.02	0.86 ± 0.11
Erucic acid	22:1n-9	MUFA	0.51 ± 0.07	0.55 ± 0.13	ND	ND
α-Linolenic acid	18:3n-3	ω-3 PUFA	2.65 ± 0.19	3.00 ± 0.20	2.26 ± 0.23	1.20 ± 0.14
Eicosapentaenoic acid	20:5n-3	ω-3 PUFA	0.40 ± 0.03	0.39 ± 0.03	0.26 ± 0.02	0.24 ± 0.05
Docosapentaenoic acid	22:5n-3	ω-3 PUFA	1.66 ± 0.07	1.76 ± 0.06	1.27 ± 0.06	1.11 ± 0.11
Docosahexaenoic acid	22:6n-3	ω-3 PUFA	0.31 ± 0.02	0.32 ± 0.02	0.24 ± 0.02	0.25 ± 0.02
Linoleic acid	18:2n-6	ω-6 PUFA	16.05 ± 1.28	16.74 ± 1.05	24.93 ± 1.07	18.61 ± 1.60
γ-Linolenic acid	18:3n-6	ω-6 PUFA	ND	ND	0.14 ± 0.01	0.11 ± 0.02
Eicosadienoic acid	20:2n-6	ω-6 PUFA	0.29 ± 0.03	0.35 ± 0.03	0.51 ± 0.03	0.33 ± 0.05
Dihomo-γ-linolenic acid	20:3n-6	ω-6 PUFA	0.38 ± 0.03	0.44 ± 0.04	0.88 ± 0.04	0.13 ± 0.07
Arachidonic acid	20:4n-6	ω-6 PUFA	2.26 ± 0.06	2.33 ± 0.07	1.91 ± 0.07	1.93 ± 0.07
Adrenic acid	22:4n-6	ω-6 PUFA	0.31 ± 0.03	0.28 ± 0.02	ND	ND

*SFA* saturated fatty acid, *MUFA* monounsaturated fatty acid, *PUFA* polyunsaturated fatty acid, *C:Dn-x* number of carbon atoms:number of double bonds-position, *ND* not detected

**Fig. 1:**
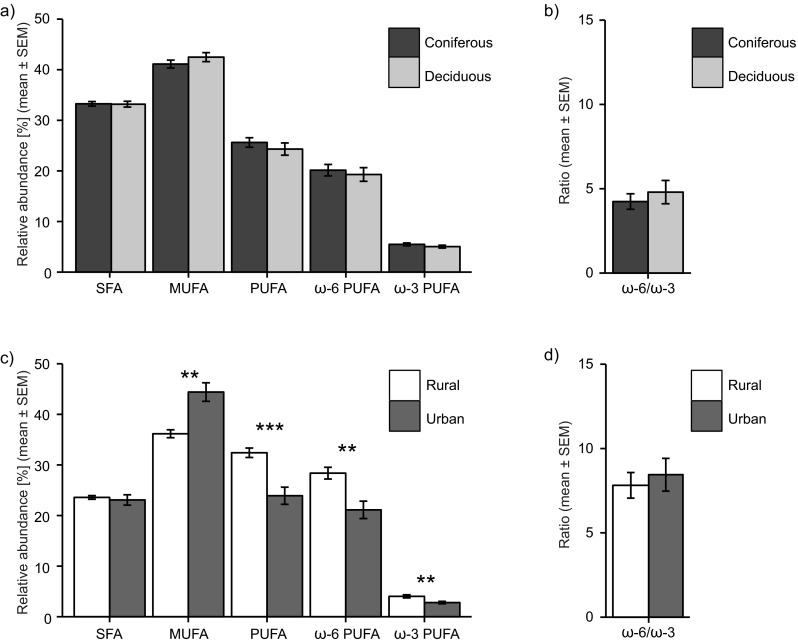
Relative abundance (% of total fatty acid content) of total saturated fatty acids (SFA), monounsaturated fatty acids (MUFA), polyunsaturated fatty acids (PUFA) as well as the ratio between total ω-6 PUFA and total ω-3 PUFA of great tit egg yolks. **a**, **b** FA composition of yolks from the deciduous and coniferous habitats in the UK. **c**, **d** FA composition of yolks from urban and rural habitats in Sweden. Significance levels are indicated by *asterisks* (*p = 0.05–0.01, **p = 0.01–0.001, ***p < 0.001)

The proportion of total SFA, total MUFA, total PUFA, total ω-3 PUFA, total ω-6 PUFA, and the total ω-6 to total ω-3 PUFA ratio did not differ significantly between the coniferous and deciduous habitat (Fig. 1a, b; see Supplementary Table 1 for additional statistical details). The levels of each individual FA were also similar in the deciduous and coniferous habitats in the UK (Table 1). In the urban–rural comparison, however, egg yolks from the urban habitat contained a significantly higher proportion of total MUFA (*F*_1,18_ = 15.34; *p* = 0.001), while yolks from the rural habitat had a higher proportion of total PUFA (*F*_1,18_ = 17.79; *p* < 0.001), including both total ω-6 PUFA (*F*_1,18_ = 12.09; *p* = 0.003) and total ω-3 PUFA (*F*_1,18_ = 12.34; *p* = 0.002) (Fig. 1c, d; see Supplementary Table 2 for additional statistical details). The difference in total MUFA between the urban and rural habitats was almost entirely due to a large difference in oleic acid, while the difference in the abundant ω-6 PUFA linoleic acid to a large extent explained the difference in both total PUFA and total ω-6 PUFA (Table 1). The habitat-related difference in total ω-3 PUFA was due to variation mainly in the proportion of α-linolenic acid (αLNA) and docosapentaenoic acid (DPA) (Table 1). There was no significant difference between yolks from the urban and rural habitats in total SFA, or in the total ω-6 PUFA to total ω-3 PUFA ratio. In the Swedish habitats, there were no significant associations between the different FA groups and the proportion of deciduous to coniferous trees or canopy cover within the territory. However, in the urban habitat, there was a trend for a negative association between the proportion of ω-6 PUFA and the percentage of deciduous trees in the close vicinity of the nest box (*F*_1,14_ = 4.16, *p* = 0.064). However, no such trend was found in the rural habitat (*F*_1,14_ = 1.59, *p* = 0.231).

**Fig. 2:**
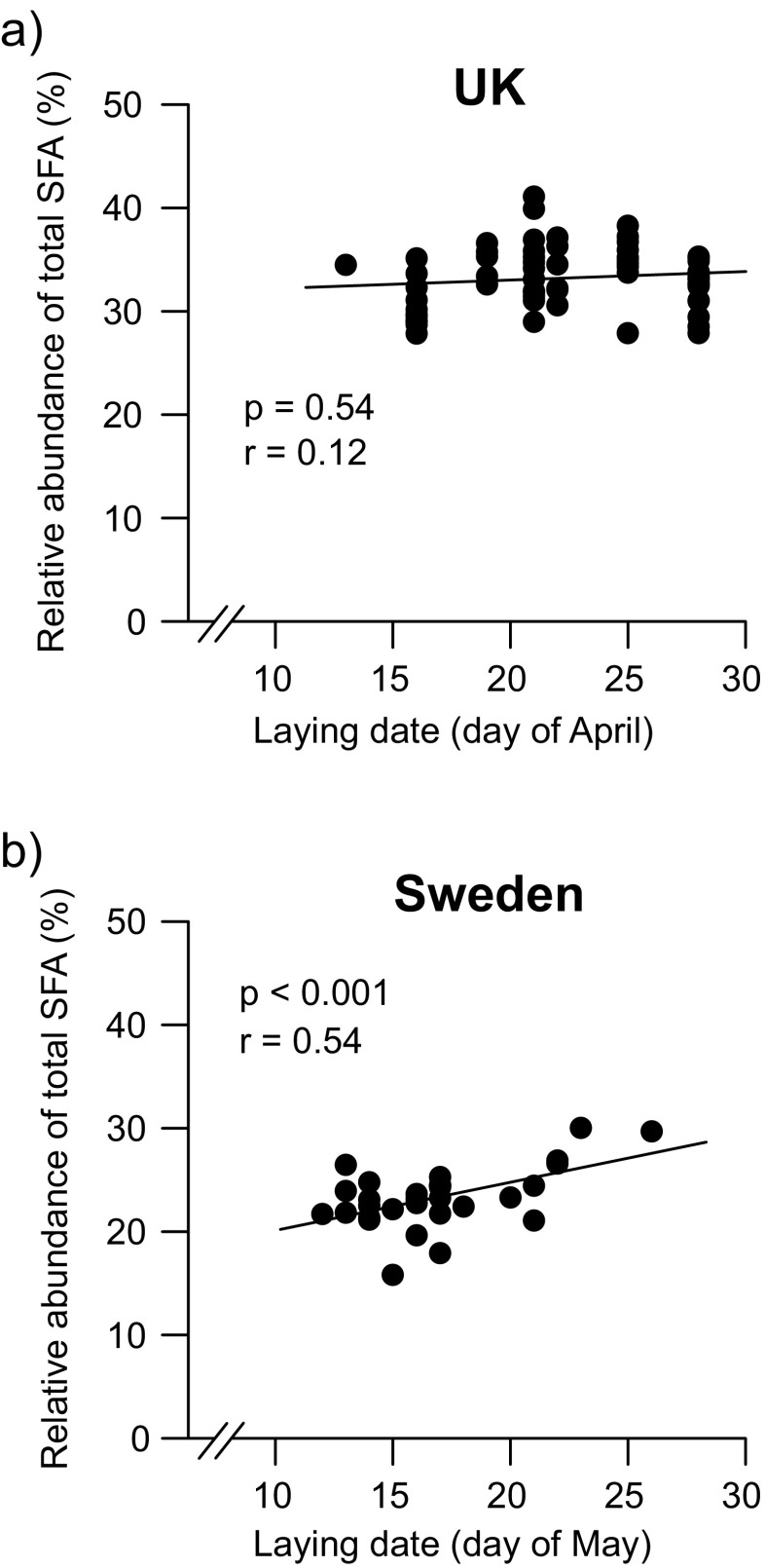
Relative abundance (% of total fatty acid content) of total saturated fatty acids (SFA) in yolks in relation to laying date across the populations from **a** the UK and **b** Sweden

Laying date was not associated with the proportion of any FA class in the British yolks across the coniferous and deciduous habitats, but significantly positively correlated with total SFA in the Swedish yolks across the urban and rural habitats (*F*_1,18_ = 15.60; *p* < 0.001, Fig. 2; Supplementary Table 1; Supplementary Table 2). The unsaturated FA classes and the total ω-6/ω-3 PUFA ratio showed no significant associations with laying date across the urban and rural populations in Sweden.

**Fig. 3:**
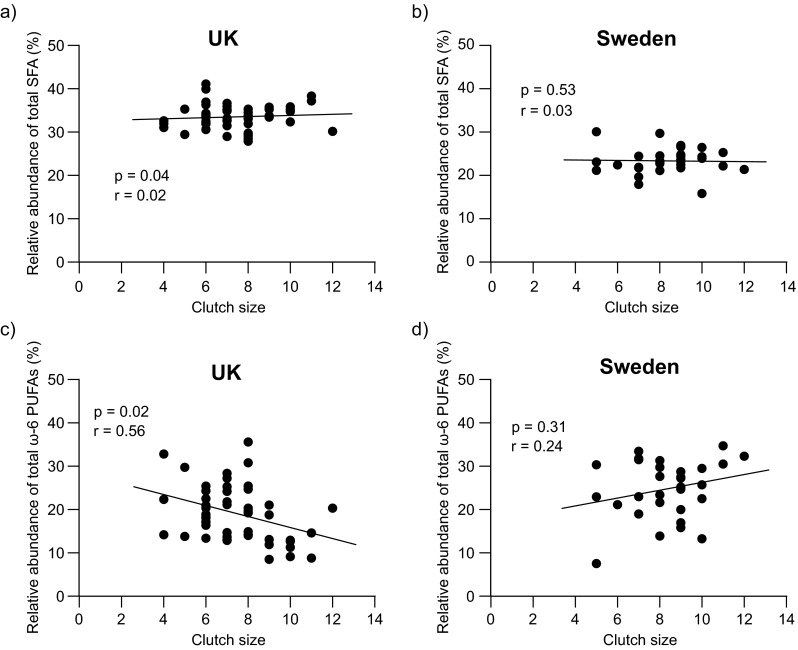
Relative abundance (% of total fatty acid content) of total saturated fatty acids (SFA) in yolks across the populations in **a** the UK and **b** Sweden in relation to clutch size. **c**, **d** Relationship between the relative abundance of total ω-6 polyunsaturated fatty acids (PUFAs) and clutch size in the same populations

In the British samples, there was no significant effect of egg number in the laying sequence on any of the FA classes or the ω-6/ω-3 ratio (Supplementary Table 1). However, clutch size was positively associated with total SFA (*F*_8,32_ = 2.40; *p* = 0.04; Fig. 3a; Supplementary Table 1) and negatively associated with total ω-6 PUFA (*F*_8,32_ = 2.7; *p* = 0.02; Fig. 3c; Supplementary Table 1) across the coniferous and deciduous habitats. In contrast, clutch size was not associated with the proportion of any FA class across the urban and rural habitats in Sweden (Fig. 3b, d, Supplementary Table 2).

**Fig. 4:**
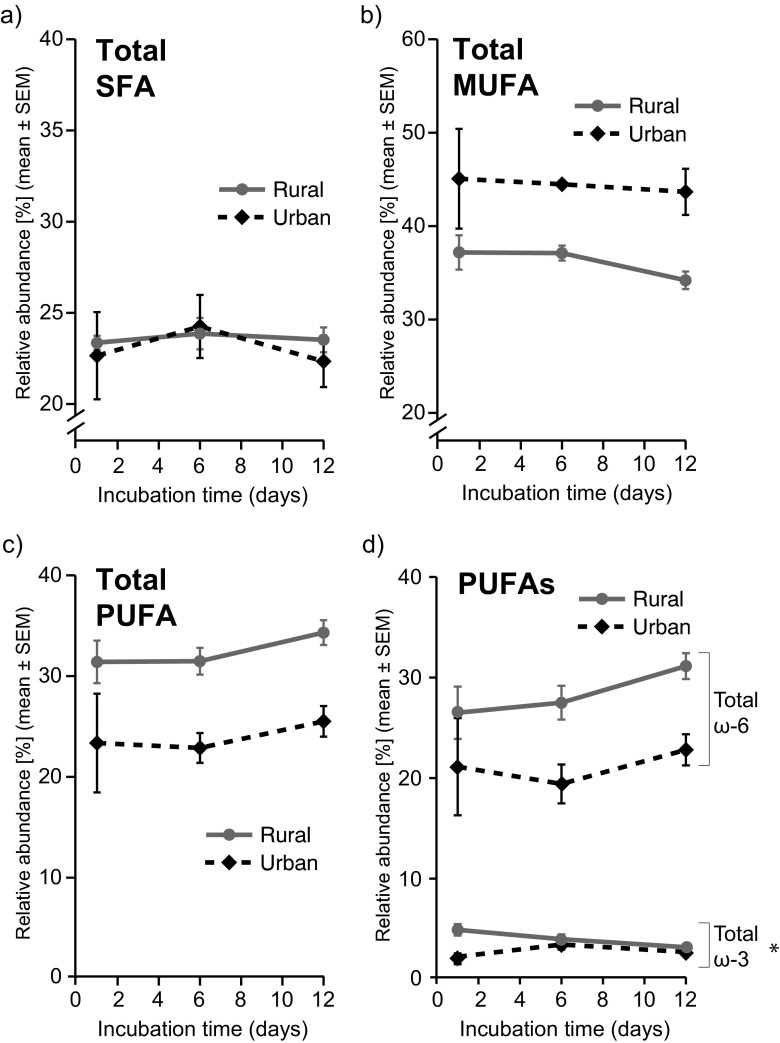
Relative abundance (% of total fatty acid content) of yolk fatty acids in relation to artificial incubation period (1, 6, and 12 days) of eggs from the urban and rural populations. **a** Total saturated fatty acids (SFA). **b** Total monounsaturated fatty acids (MUFA). **c** Total polyunsaturated fatty acids (PUFA). **d** Total ω-6 PUFA and total ω-3 PUFA. The *asterisk* in **d** indicates the significant interaction between habitat (urban or rural) and incubation time (see main text for details) for total ω-3 PUFA

In order to address whether or not incubation time affects the FA composition of yolk, the Swedish eggs were artificially incubated for three different time periods (1, 6, and 12 days; Fig. 4). In the simplified statistical model (i.e., when excluding laying date and clutch size from the model), there was a significant interaction between the fixed factors “habitat” (urban or rural) and “incubation time” (1, 6, or 12 days) for the proportion of total ω-3 PUFA (*F*_2,24_ = 2.40; *p* = 0.02; Fig. 4d), indicating a decline over time in the rural but not the urban habitat. Specifically, the proportion of ω-3 PUFA was the highest in the rural yolks during the first incubation day, but then similar in both populations at days 6 and 12 (Fig. 4d). None of the other FA classes, nor the total ω-6 to total ω-3 ratio, differed significantly between the eggs that were incubated during different numbers of days in the two habitats (*F*_5,24_ = 0.02–1.69, *p* = 0.21–0.98).

## Discussion

This is the first study to report variation in egg yolk FA composition in the context of urban and rural habitats, and one of the first to investigate yolk FA differences in relation to the habitat in which eggs are laid (Bourgault et al. 2007). Our results show that the relative abundance of all FA classes, except for SFA, differed between great tit yolks from the urban and the rural habitats, but none of the FA classes differed significantly between the coniferous and deciduous habitats or territories. Specifically, the urban yolks had a higher proportion of total MUFA and lower proportions of both ω-3 PUFA and ω-6 PUFA compared with the rural yolks. Interestingly, the fact that the coniferous and deciduous habitats in the UK are more different in terms of dominating vegetation than the urban park and rural forest in Sweden suggest that factors associated with the urban habitat other than vegetation type (except for perhaps ω-6 PUFA in the urban habitat where a trend for a negative association with percentage of deciduous trees was found) underlie the observed between-habitat differences in FA composition of great tit egg yolk.

### Possible effects of different diets

Due to the higher abundance of supplementary foods such as seeds and nuts in the urban habitat, we predicted that urban yolks would contain a higher proportion of the MUFA oleic acid and the ω-6 PUFA linoleic acid, which dominate the FA profile of these foods. Our results are in line with this prediction for oleic acid (Table 1), but not for linoleic acid, which was more abundant in the rural yolks. Since linoleic acid is strictly dietary, this result is likely to reflect differences in maternal nutrition between the urban and rural habitat. Thus, in contrast to our prediction, this result indicates that the diet composition in the urban habitat does not increase, but rather decreases, the relative linoleic acid content of yolk, compared with the food items eaten by rural females.

Previous studies on a variety of bird species have shown that supplementing the maternal diet with ω-3 PUFA enhances the ω-3 PUFA content of yolk (Nash et al. 1995; Surai et al. 2001). Since the availability of insects as food, such as overwintering pupae that contain high levels of the essential ω-3 PUFA αLNA (Andersson et al. 2015), might be higher in the rural forest than in the urban habitat, we predicted that rural yolks would have a relatively higher proportion of ω-3 PUFA. Our results are in line with this prediction. However, in contrast to Bourgault et al. (2007), we did not find any differences in yolk FA composition between the different “natural” habitats (in our case between coniferous and deciduous forests). While this result was somewhat surprising, it suggests that the present coniferous and deciduous forests might offer similar foods for great tit females during egg formation (i.e., at least similar in terms of FA composition), as compared to the deciduous and evergreen oak habitats that were used to study FA variation in eggs of the blue tit (Bourgault et al. 2007). Another possibility is that the great tits from the coniferous habitat (in the UK) might leave their territories to forage elsewhere, and possibly from the same food sources that the birds from the deciduous plots have access to. However, this explanation might be less likely due to the fact that great tits generally forage within their territories during breeding season (Perrins 1991). Differences in relative levels of certain FAs (i.e., αLNA and arachidonic acid) were previously found in the plasma of 15-day-old nestlings from the exact same coniferous and deciduous habitats and sampling year (Isaksson et al. 2015). However, since plasma is highly and rapidly affected by the FA content of the diet (Hulbert and Abbott 2012; M.N. Andersson and C. Isaksson et al., unpublished data), and since nestlings are mainly fed caterpillars that have a different FA content as compared to the seed-dominated diet available to females during egg formation, no such correlation would be expected.

In addition to possible differences in diets, differential maternal allocation of FAs to the yolk could also contribute to the different FA compositions between urban and rural eggs (Sandell et al. 2007). In this scenario, the rural females may afford to invest in higher quality eggs as compared to urban females, because they might be less constrained in terms of dietary availability of essential and physiologically important PUFAs. If their own requirements are fulfilled, they may be able to invest in higher quality eggs/offspring, whereas the urban females may selectively retain the PUFAs for their own use at the expense of egg/offspring quality, i.e., similar to what has been found for hormones (Sandell et al. 2007). However, our previous studies on FA composition of female plasma in both the Swedish and British populations show variations in FA composition that are similar to the patterns found here for yolk, supporting the dietary-based explanation rather than differential allocation (Andersson et al. 2015; Isaksson et al. 2015). Indeed, the urban birds in Sweden showed a trend both for higher relative oleic acid levels (thus also total MUFA) and lower relative levels of the major ω-6 and ω-3 PUFAs, compared with the rural birds (Andersson et al. 2015), which is similar to what we found in the yolks in the present study.

### Possible consequences for the hatched chick

Differences in early-life nutrition can have physiological carry-over effects on the hatched chick, with both short- and long-term effects on survival and reproductive behavior (Lindström 1999; Metcalfe and Monaghan 2001). However, little is known about potential effects of yolk micronutrient composition, such as the FA profile, on later-life phenotypes, although some lessons can be learned from previous studies on fowl. Indeed, supplementation of ω-3 PUFA to poultry diet prior to egg formation was more effective than post-hatching supplementation of offspring diet in enhancing cardiac tissue ω-3 PUFA status of the poultry chick, which is likely associated with improved health (Cherian 2007). Indeed, chicks hatched from the ω-3 PUFA-provided hens produced less pro-inflammatory eicosanoids (derived from arachidonic acid) than chicks from hens fed a low ω-3 PUFA diet—a physiological status that is associated with reduced risk of metabolic and inflammatory-related disorders in poultry (Cherian 2007). In addition, an increase in oleic acid (the dominant MUFA of yolk) and simultaneous decrease in the ω-3 PUFA eicosapentaenoic acid was previously observed in poultry that died due to sudden death syndrome (Cherian 2007, and references therein). If these relationships also occur in altricial species (cf. Metcalfe and Monaghan 2001) such as the great tit is unknown, however assuming that they do, the higher MUFA level in the urban eggs of the present study in combination with their lower levels of ω-3 PUFAs suggest that they are subjected to increased risks for certain health disorders compared to their rural conspecifics; thus, they might have lower survival.

However, the statistical interaction that was found between habitat type and artificial incubation time for ω-3 PUFA suggests that embryos may selectively and rapidly utilize or compensate for different initial levels of ω-3 PUFA in yolk since the habitat difference is only evident at day 1 of incubation (see also Lin et al. 1991). At 6 and 12 days of artificial incubation, the proportion of ω-3 PUFA was the same in both urban and rural yolks. Differences between urban and rural yolks among all other FA classes remained the same across the range of sampled incubation stages, indicating that the habitat-related differences in FA composition are robust.

### Effects of laying date

In Sweden, laying date was positively associated with the relative level of total SFA across both habitats. This might be a result of changes in ambient temperatures as the spring progresses, i.e., the relative level of total SFA in animal tissue generally increases with ambient temperature (Yom-Tov and Tietz 1978; Andersson et al. 2015; M.N. Andersson and C. Isaksson et al. unpublished data). Indeed, daily average temperatures increased relatively rapidly along the sampling period in Sweden, but to a lesser extent in the UK (Supplementary Table 3). Since SFA can be synthesized de novo, it is possible that females increase the biosynthesis of SFA or mobilize more of their stored SFA to the yolk as ambient temperature increases as the season progresses. Due to the high melting point of SFA, a high proportion of yolk SFA during early spring may lead to more rigid cell membranes (i.e., lower membrane fluidity) in the embryo, which affects several membrane functions such as the flux of nutrients and oxygen (Sinensky 1974; Hazel 1995). Alternatively, the increase in relative SFA in yolks with laying date could be an effect of female quality or condition; indeed, different SFA proportions in adult plasma were previously shown to be associated with body condition in both of the studied population pairs (Andersson et al. 2015; Isaksson et al. 2015). Good quality and/or experienced females breed earlier in the season than females in poorer condition, and/or those that are inexperienced (Verhulst et al. 1995). Thus, condition-dependent breeding times could possibly underlie the association between yolk SFA and laying date.

### Trade-off between egg quantity and quality?

Finally, food, as well as specific nutrients, is often a limited resource for wild animals, which can constrain reproductive investment either via the number or quality of offspring (Smith et al. 1989; Blount et al. 2004; Williams 2005). Regarding FAs, particularly the ɷ-3 PUFAs are generally in short supply for great tits (Andersson et al. 2015). FAs of this class have crucial functions throughout life; early in life, they are needed for proper development of several tissues, whereas later in life, they serve important roles as signaling molecules and by mediating anti-inflammatory responses (Larsson et al. 2004; Hohl and Rosen 1987; Pavoine et al. 1999). As egg production is nutritionally demanding (Williams 2005), a classical trade-off between egg number and egg quality in terms of ω-3 PUFA content could be expected, with eggs from larger clutches containing a lower proportion of ω-3 PUFA compared to eggs from smaller clutches. However, our results from the British populations showed that the proportion ω-6 PUFA, rather than that of ω-3 PUFA, was negatively associated with clutch size, whereas the proportion of SFA was positively associated with clutch size. Interestingly, the yolks from the British populations contained much less of the major ɷ-6 PUFA linoleic acid than the Swedish populations (on average 16 % compared to 22 %, in the British and Swedish populations, respectively; Table 1). Thus, despite the fact that ω-6 PUFAs are present in much higher proportions than ω-3 PUFAs, it is possible that they are a limited resource in certain environments, such as the British forests, whereas the higher abundance in the Swedish populations might emancipate a potential trade-off between egg quality and quantity. Future studies should experimentally manipulate dietary ω-6 PUFA for egg-laying females to test whether this association reflects a true constraint.

In contrast to our prediction, there was no association between ω-3 PUFAs (or any other FA class) and egg number in the laying sequence, suggesting that females partition their FAs equally among all eggs in the clutch. However, it is unknown whether the proportion of PUFAs is reduced in the last eggs in the laying sequence, because these eggs were not analyzed.

### Conclusions and future directions

Taken together, this study indicates that habitat-related differences in yolk FA composition can be linked to factors associated with urban habitats rather than differences in dominating habitat vegetation. Future studies should investigate whether egg yolks from additional urban/rural habitat pairs show similar differences in their FA content, and whether the observed differences are consistent between years and across spatial scales. Future studies should also aim to unravel whether the observed differences between the urban and rural yolks have any implications for chick development and whether anthropogenic food sources indeed are the causal mechanism for the difference. Studies addressing the physiological mechanisms that influence individual and population level fitness are crucial for understanding how urban habitats affects bird communities and how we potentially could reduce human impact on wildlife.

## Electronic supplementary material

ESM 1 (DOCX 23 kb)

ESM 2 (XLSX 47 kb)
