# Supplementary material for: Fatty acid profiles of great tit (Parus major) eggs differ between urban and rural habitats, but not between coniferous and deciduous forests
Source: Naturwissenschaften. 2016 Jun 14;103:55. doi: 10.1007/s00114-016-1381-0 (PMC4908168; doi:10.1007/s00114-016-1381-0)
Supplement: Supplementary file 1 — (DOCX 23 kb) [file 114_2016_1381_MOESM1_ESM.docx]

**Supplementary file**

Journal: The Science of Nature – Naturwissenschaften

**Fatty acid profiles of great tit (*Parus major*) eggs differ between urban and rural habitats, but not between coniferous and deciduous forests**

Alejandra Toledo^1,2^, Martin N. Andersson^1^, Hong-Lei Wang^1^, Pablo Salmón^1^, Hannah Watson^1^, Graham C. Burdge^3^, Caroline Isaksson^1^*

^1^*Department of Biology, Lund University, Lund, Sweden*

^2^Current address: *Department of Life Science, University of Roehampton, London, United Kingdom*

^3^*Academic Unit of Human Development and Health, Faculty of Medicine, University of Southampton, United Kingdom.*

* Corresponding author: [Caroline.Isaksson@biol.lu.se](mailto:Caroline.Isaksson@biol.lu.se)

**Supplementary Table 1.** Summary statistics for the fatty acid composition of great tit egg yolks from the coniferous and deciduous habitats in the United Kingdom. Laying date, clutch size and laying order was included as covariates. Significant effects are highlighted in bold.

|  |  | **Habitat** | **Laying** | **Clutch** | **Laying** |
| --- | --- | --- | --- | --- | --- |
|  |  |  | **date** | **size** | **order** |
| **SFA** | **SS** | < 0.01 | < 0.01 | 0.043 | < 0.01 |
|  | ***F*** | 0.04 | 0.37 | 2.40 | 0.63 |
|  | ***p*** | 0.84 | 0.55 | **0.04** | 0.43 |
| **MUFA** | **SS** | 0.02 | < 0.01 | 0.09 | < 0.01 |
|  | ***F*** | 3.73 | 0.48 | 1.73 | 0.47 |
|  | ***p*** | 0.06 | 0.49 | 0.13 | 0.50 |
| **PUFA** | **SS** | 0.05 | < 0.01 | 0.34 | < 0.01 |
|  | ***F*** | 2.99 | < 0.01 | 2.76 | 0.03 |
|  | ***p*** | 0.09 | 0.98 | **0.02** | 0.86 |
| **ω-6 PUFA** | **SS** | 0.07 | < 0.01 | 0.59 | < 0.01 |
|  | ***F*** | 2.63 | 0.15 | 2.70 | 0.20 |
|  | ***p*** | 0.11 | 0.70 | **0.02** | 0.66 |
| **ω-3 PUFA** | **SS** | < 0.01 | 0.04 | 0.14 | < 0.01 |
|  | ***F*** | < 0.01 | 3.16 | 1.38 | 0.29 |
|  | ***p*** | 0.95 | 0.08 | 0.24 | 0.59 |
| **ω-6/ω-3** | **SS** | 0.05 | 0.05 | 0.72 | 0.01 |
|  | ***F*** | 1.08 | 1.18 | 2.04 | 0.32 |
|  | ***p*** | 0.30 | 0.29 | 0.07 | 0.57 |

Abbreviations: SFA = saturated fatty acid, MUFA = monounsaturated fatty acid, PUFA = polyunsaturated fatty acid, and SS = Sum of Squares.

**Supplementary Table 2.** Summary statistics for the fatty acid compositions in great tit egg yolks from the urban and rural habitats in Sweden. Significant effects are highlighted in bold.

|  |  | **Habitat** | **Incub. stage** | **Laying date** | **Clutch size** |
| --- | --- | --- | --- | --- | --- |
| **SFA** | **SS** | < 0.01 | < 0.01 | 0.06 | 0.02 |
|  | ***F*** | 0.60 | 0.67 | 15.6 | 0.89 |
|  | ***p*** | 0.45 | 0.53 | **< 0.001** | 0.52 |
| **MUFA** | **SS** | 0.17 | < 0.01 | < 0.01 | 0.06 |
|  | ***F*** | 15.34 | 0.43 | 0.45 | 0.76 |
|  | ***p*** | **0.001** | 0.66 | 0.51 | 0.63 |
| **PUFA** | **SS** | 0.29 | 0.03 | 0.02 | 0.15 |
|  | ***F*** | 17.79 | 0.65 | 1.43 | 1.31 |
|  | ***p*** | **< 0.001** | 0.40 | 0.25 | 0.30 |
| **ω-6 PUFA** | **SS** | 0.27 | 0.08 | 0.05 | 0.20 |
|  | ***F*** | 12.09 | 1.33 | 2.07 | 1.28 |
|  | ***p*** | **< 0.01** | 0.29 | 0.17 | 0.31 |
| **ω-3 PUFA** | **SS** | 0.22 | 0.05 | 0.04 | 0.17 |
|  | ***F*** | 12.34 | 1.32 | 2.08 | 1.37 |
|  | ***p*** | **< 0.01** | 0.29 | 0.17 | 0.28 |
| **ω-6/ω-3** | **SS** | < 0.01 | 0.14 | 0.14 | 0.34 |
|  | ***F*** | 0.08 | 2.12 | 4.16 | 1.45 |
|  | ***p*** | 0.77 | 0.15 | 0.06 | 0.25 |

Abbreviations: SFA = saturated fatty acid, MUFA, monounsaturated fatty acid, PUFA = polyunsaturated fatty acid, and SS = Sum of Squares.
